# Global Prevalence of Internet Gaming Disorder: An Umbrella Review of Meta-Analytic Evidence and Implications for Child and Adolescent Psychiatry

**DOI:** 10.3390/brainsci16070728

**Published:** 2026-07-09

**Authors:** Elisabeth C. S. Poon, Xun Ci Soh, Trina J. H. Poh, Andre C. S. Tan, Andree Hartanto

**Affiliations:** School of Social Sciences, Singapore Management University, Singapore 179873, Singapore; cs.poon.2023@socsc.smu.edu.sg (E.C.S.P.);

**Keywords:** internet gaming disorder, IGD, prevalence, umbrella review

## Abstract

**Highlights:**

**What are the main findings?**

- Median IGD prevalence was 6.20% (IQR: 4.25% to 8.30%).
- Subgroup findings were mixed across meta-analyses.

**What are the implications of the main findings?**

- IGD affects a considerable proportion of the population.
- Greater methodological consistency is needed.

**Abstract:**

Background/Objectives: Internet gaming has become a widespread global activity, raising concerns about the prevalence of Internet Gaming Disorder (IGD). However, existing meta-analyses report highly variable prevalence estimates. This umbrella review aims to clarify the prevalence and variability of IGD by synthesizing evidence from meta-analyses to provide a comprehensive overview of IGD prevalence across populations, age groups, and regions. Methods: Following PRISMA guidelines, systematic searches were carried out across five published literature databases and two supplementary search sources. Title and abstract screening, followed by full-text eligibility assessment, were conducted independently by three authors. The same three authors then performed data extraction and quality assessment for the nine included meta-analyses. Results: After screening, nine eligible meta-analyses were included. The overall median prevalence of IGD was 6.20%, with an interquartile range of 4.25% to 8.30%. Subgroup analyses indicated a higher prevalence among males compared to females. Evidence for age differences between adolescents and adults was limited and should be interpreted cautiously because age groups were inconsistently defined across meta-analyses. Regional comparisons suggested higher prevalence estimates in Asia than in Europe, and gamer profile comparisons suggested higher prevalence estimates in gamers than in the general population, although evidence for these differences was limited and not consistent across meta-analyses. Conclusions: Overall, the findings indicate that IGD affects a sizeable proportion of the population, but prevalence estimates vary depending on demographic and regional characteristics. Greater consistency in diagnostic criteria, assessment tools, and reporting standards is needed to improve the comparability and interpretability of IGD prevalence research.

## 1. Introduction

Over the past two decades, internet gaming has become a widespread form of entertainment and social interaction. Its popularity intensified during the COVID-19 pandemic, when online games provided opportunities for social connection during periods of physical distancing and lockdowns [1,2,3,4]. At the same time, technological advances in broadband infrastructure, mobile connectivity, and digital gaming platforms have made internet gaming increasingly accessible across personal computers, consoles, and smartphones. Recent estimates suggest that there are 2.87 billion video game players globally, with Asia comprising the largest share of active gamers worldwide [5]. Given the scale of gaming participation, even a relatively small proportion of individuals experiencing problematic gaming may represent a substantial public health concern.

Although gaming is often recreational and may be associated with positive outcomes, excessive or poorly regulated gaming has been linked to several adverse consequences. Moderate gaming has been associated with benefits such as improved mood, self-esteem, visuo-spatial skills, and processing speed [6,7,8,9,10]. However, these effects may not be linear. For example, one study found that while gaming time was positively associated with executive and social cognitive performance at moderate levels, longer gaming time was associated with poorer outcomes [6]. This pattern is consistent with the “Goldilocks hypothesis,” which proposes that moderate digital engagement may be neutral or beneficial, whereas excessive use may be harmful [11,12]. Problematic gaming has also been associated with poor sleep, impaired concentration, somatic complaints, reduced social engagement, task neglect, and academic, professional, or interpersonal difficulties [13,14,15,16,17,18]. These findings highlight the need to better understand the extent to which problematic gaming occurs across populations.

Clinical interest in problematic gaming has increased alongside its recognition in major diagnostic frameworks. The Diagnostic and Statistical Manual of Mental Disorders, Fifth Edition (DSM-5), included Internet Gaming Disorder (IGD) as a condition for further study, while the International Classification of Diseases, Eleventh Revision (ICD-11), formally recognized Gaming Disorder (GD) as a diagnosis [19,20]. While ICD-11 includes an offline subtype, this falls outside the main focus of our umbrella review, which focuses specifically on online or internet-based gaming. Although the DSM-5 and ICD-11 differ in terminology and diagnostic structure, both frameworks emphasize impaired control over gaming, increasing priority given to gaming over other activities, and continuation of gaming despite negative consequences. In the present review, IGD is used to refer broadly to clinically relevant problematic gaming as assessed by DSM-5- or ICD-11-informed criteria and instruments.

Despite growing research attention, the prevalence of IGD remains difficult to summarize. Existing reviews have reported a wide range of estimates. For example, a study found that prevalence estimates ranged from 0.70% to 27.50% across epidemiological studies [21]. More recent meta-analyses have reported pooled estimates such as 3.05% globally, with higher estimates in Asia than in Europe [22], and 6.70% when studies based on DSM-5 and ICD-11 criteria were synthesized [23]. These differences are likely influenced by variation in assessment instruments, diagnostic criteria, sample characteristics, age groups, gamer versus general-population samples, and geographic coverage. As a result, it remains challenging to determine the overall magnitude of IGD prevalence and to identify which factors are most consistently associated with higher prevalence estimates. Adolescence is a clinically relevant developmental period for IGD because gaming occurs alongside ongoing maturation of self-regulation and reward sensitivity and is frequently accompanied by broader psychosocial difficulties such as peer problems, perceived stress, and hyperactivity [24,25]. Therefore, prevalence estimates in younger populations should be interpreted not only as epidemiological figures but also in relation to developmental contexts such as sleep, school functioning, family conflict, and peer relationships.

Therefore, this umbrella review aims to address this gap by synthesizing meta-analytic prevalence estimates across diverse populations and regions to examine how demographic and cultural factors shape these estimates while also considering implications for younger populations where relevant. Specifically, this review summarizes the overall prevalence estimates reported in prior meta-analyses, examines subgroup findings where available, including gender, age, region, gamer profile, and assessment tool, and evaluates the methodological quality of the included meta-analyses. By synthesizing the highest tier of evidence, the current review seeks to clarify the magnitude and variability of IGD prevalence and inform future research, prevention efforts, and public health responses.

## 2. Materials and Methods

### 2.1. Transparency and Openness

This current umbrella review was pre-registered under PROSPERO at https://www.crd.york.ac.uk/PROSPERO/view/CRD420251115847. For transparency, amendments to the PROSPERO record and review materials made after registration are acknowledged here. We made all review materials available on OSF, including the inclusion criteria, title-and-abstract screening sheet, full-text screening sheet, quality assessment sheet, data extraction sheets, and the PRISMA 2020 checklist (Table S1) (https://osf.io/6pkna/overview?view_only=9a13d4018f73453fbec2f4260b950e20). A second version of the data extraction sheet was uploaded because the initial version did not include the number of studies contributing to each subgroup analysis. Furthermore, this review adhered to the Preferred Reporting Items for Systematic Reviews and Meta-Analyses (PRISMA) guidelines [26]. Following record retrieval, duplicate records were manually removed using Zotero (version 7.0.15, Vienna, VA, USA) [27].

### 2.2. Search Strategy

Systematic searches were performed by three authors across various resources to retrieve meta-analyses. An initial search was conducted on 1 August 2025 across five published literature databases (EBSCOhost ERIC, EBSCOhost PsycINFO, PubMed, Web of Science, and Scopus) to identify peer-reviewed records. In addition, two supplementary search sources (ProQuest Dissertations & Theses Global; Google Scholar) were also included for gray literature to minimize publication bias and ensure a more thorough overview of the topic [28]. Results were sorted by relevance, and the first 250 records were retrieved from each source without additional filters, thereby ensuring comprehensive coverage of both published and gray literature [29]. Keywords from the abovementioned databases are shown in Table 1.

A simplified search string was used for Google Scholar as it does not support truncation operators [30]. Moreover, due to a character limit of 256 characters, it is impossible to use a longer search string to capture relevant records [31]. All records from the five databases and the first 250 records from the additional two sources were retrieved for further screening. Duplicates were automatically identified using Zotero and manually checked and merged by the third author.

### 2.3. Selection Criteria

The eligibility of meta-analyses for inclusion in this umbrella review was determined independently by the first, third, and fourth authors in three stages. In the first stage, the titles and abstracts of extracted records were screened for relevance based on inclusion criteria. In the next stage, the remaining records were evaluated based on full texts. Lastly, quality assessment and data extraction were carried out for the records that met all the criteria. A trial screening was conducted for each stage, where approximately 10.00% (i.e., 173 for the first stage and 18 for the second stage) and 55.00% (i.e., 5 for the third stage) of the total records were screened to ensure authors reached a consensus regarding the inclusion criteria. All eligibility criteria were established to ensure that the included records met a minimum standard of methodological rigor and addressed the same core construct. Specifically, we consistently identified meta-analyses that included a systematic search component and reported IGD prevalence as a quantitative outcome.

Titles and abstracts were screened using the following criteria:

1. Records were included if they were written or otherwise documented in English.
2. Records were included if they were meta-analyses with a systematic search component (i.e., quantitative systematic reviews).
3. Records were included if they mentioned Internet Gaming Disorder (IGD).

Interrater agreement rates for trial screening of titles and abstracts were 97.00%, 78.00%, and 80.90% for the first, second, and third criteria, respectively. The remaining 1560 records were subsequently screened, and the interrater agreement rates were 99.70%, 87.10%, and 83.70% for each respective criterion. For both screenings, disagreements were resolved through discussion between all three authors.

Subsequently, the full texts of the 171 records were screened using additional criteria beyond those previously applied:

1. Records were included if they were published within the last 12 years (i.e., 1 January 2013 to 1 January 2025) [20].
2. Meta-analyses were included if they estimated prevalence as percentages (%).
3. For Internet Gaming Disorder, meta-analyses were included if they included studies that assessed the prevalence of clinically diagnosed disorders using at least one of the following:
  - A clinician-administered instrument and/or diagnostic interview such as The Structured Clinical Interview for DSM-5 (SCID-5) [32] and/or The Structured Clinical Interview for ICD-11 (SCII-11) [19].
  - A self-reported inventory that is guided by the DSM-5 diagnostic criteria, such as IGDT-10 [33] and/or IGD20 [34] and/or IGDS9SF [35], and/or guided by the ICD-11 criteria, such as GDT [36].

“Clinically diagnosed disorder” in criterion 3 refers to clinician-administered diagnostic interviews and self-report instruments that operationalize these criteria to identify probable or screening-positive cases, rather than a formal clinical diagnosis. Additionally, selection criteria placed no restrictions on peer review status, country, geographic region, or the timing of the COVID-19 pandemic. Consequently, the included meta-analyses could assess prevalence rates from before, during, or after the pandemic. Meta-analyses published within the 12 years following 2013 were included, as IGD was first included in the DSM-5 in that year.

Table 2 shows the raw agreement rates for trial and post-trial full-text screening. Disagreements in both screenings were resolved through discussion among the three authors.

### 2.4. Assessment of Methodological Quality

Quality assessment of included meta-analyses was done using the Joanna Briggs Institute (JBI) critical appraisal instrument for Systematic Reviews and Research Syntheses [37]. Five records were used for trial screening, followed by the remaining four records for the post-trial screening.

The critical appraisal instrument consists of 11 items that were rated “yes”, “no”, “unclear” or “not applicable” based on the following: (1) a clear and explicit research question, (2) appropriate inclusion criteria for the review question, (3) an appropriate search strategy, (4) adequate sources used to search for studies, (5) appropriate appraisal criteria for studies, (6) critical appraisal conducted by two or more reviewers independently, (7) methods used to minimize errors in data extraction, (8) appropriate methods used to combine studies, (9) assessment of the likelihood of publication bias, (10) whether recommendations for policy and/or practice were supported by the data obtained in the study, and (11) appropriate directives for future research. An overall score was derived for each meta-analysis by summing the number of “yes” responses across the 11 items. Raw percentage agreement rates for each checklist item are presented in Table 3.

### 2.5. Data Extraction

The three authors independently extracted the following data from the meta-analyses: countries and regions covered, total number of studies, total sample size, assessment tools used (i.e., clinician-administered [e.g., DSM-5] or self-reported [e.g., IGDS9-SF]), overall IGD prevalence, confidence intervals, statistical model used to synthesize the overall prevalence (i.e., random-effects model or fixed-effects model), and any subgroup analyses reported. Confidence intervals were extracted where reported, and their absence was attributed to unreported estimates in the respective meta-analyses. All three authors reviewed all the records for both the trial screening of the five meta-analyses and the post-trial screening consisting of four meta-analyses.

Overall, the average agreement rates for the trial screening were 86.15%, and for the subsequent post-trial screening, they were 82.69%. All discrepancies were resolved through discussion between the three authors; where consensus could not be reached, the second author made the final determination. Further details about the raw agreement rates of each criterion are presented in Table 4.

### 2.6. Data Synthesis

In this umbrella review, the overall prevalence of IGD was synthesized narratively. Various subgroup analyses were examined to further understand the factors associated with prevalence rates, including sample characteristics and different IGD measurement scales used across studies. Furthermore, these analyses were used to draw conclusions about gender, the type of gaming profile, time period (i.e., during, before, or post COVID-19, or even the absence of a pandemic), geographical location, age, measurement tool(s), and sampling method(s). These analyses were only conducted when at least two meta-analyses were available. As this review was narrative in nature, the findings were summarized descriptively. An overall median prevalence with IQR was reported. Using the median provides a robust summary of central tendency that is less influenced by extreme prevalence values. The median in this review is used as a descriptive review-level summary, not as a statistically optimal pooled prevalence estimate. Reporting the IQR describes the spread of the middle 50% of prevalence estimates (from the 25th to the 75th percentile), highlighting variability in typical values while reducing the influence of outliers.

## 3. Results

### 3.1. Search and Eligibility

**Figure 1:**
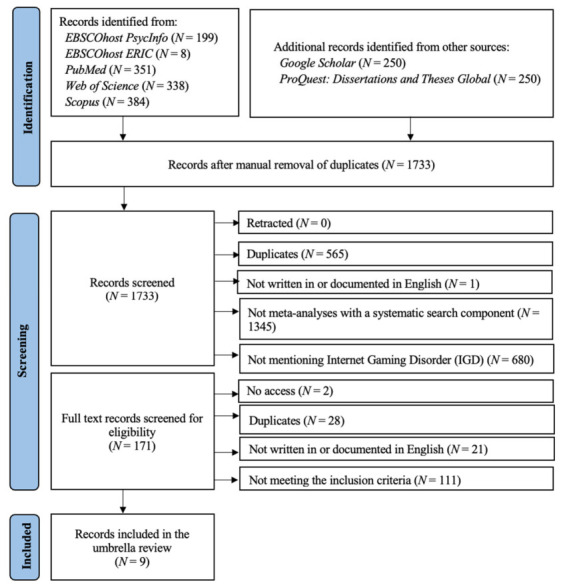
PRISMA diagram detailing study selection and reasons for exclusion. *Note.* These criteria are not mutually exclusive.

A total of 1780 records were retrieved from the search strategy, and after manual de-duplication, 1733 records were screened based on abstracts and titles, as shown in Figure 1. Overall, nine records were included in the current umbrella review.

**Figure 2:**
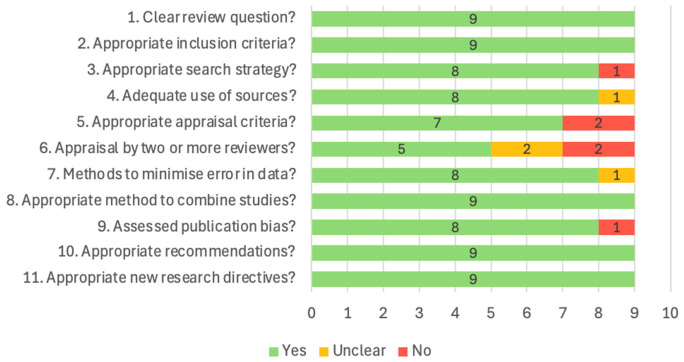
Summary of each criterion using the Joanna Briggs Institute Critical Appraisal Checklist [37].

The Joanna Briggs Institute (JBI) critical appraisal instrument was selected because it aligns with our focus on the methodological quality of systematic reviews and has been widely used in similar syntheses. The total score for each meta-analysis ranged from 9 to 11 (*Mdn* = 10, as shown in Table 5). The summed JBI score is reported for descriptive purposes only and should not be interpreted as implying that all domains carry equal methodological weight; domains such as search strategy, publication-bias assessment, and statistical synthesis are considered particularly critical. We found that item six (i.e., “critical appraisal by two or more reviewers independently”, presented in Figure 2) had the lowest number of “yes” indicated. This finding highlights the need for independent critical appraisal by at least two reviewers in future meta-analyses.

### 3.2. Review Characteristics

**Figure 3:**
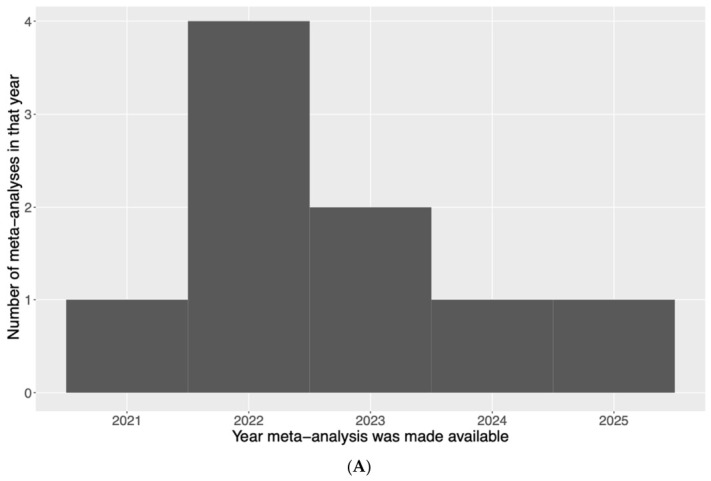

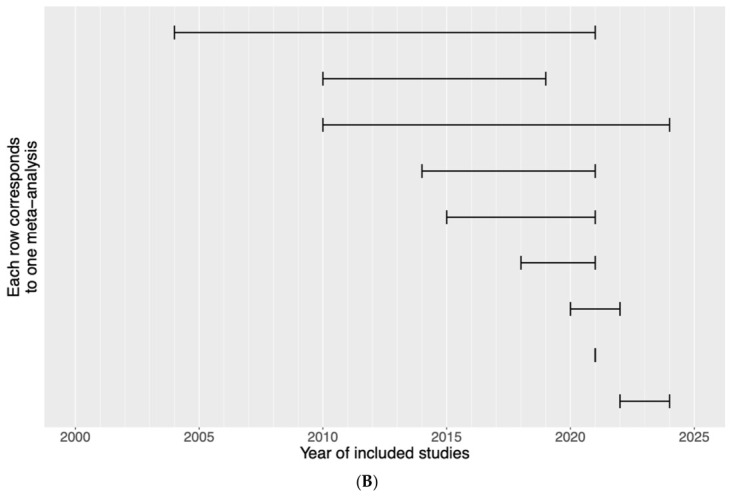
(**A**) Characteristics of included meta-analyses (year of meta-analysis). (**B**) Characteristics of included meta-analyses (year of studies in meta-analysis).

Table 6 shows the characteristics of the nine included meta-analyses. Sample sizes for the included meta-analyses ranged from 2236 to 641,763 (*Mdn* = 75,869), with all nine meta-analyses published between 1 January 2021 and 1 January 2025 (Figure 3A). Of the nine meta-analyses, three examined gamers and the general population [39,42,44], three compared adolescents and adults [23,40,44], and three compared prevalence across genders [22,39,40].

### 3.3. Prevalence of IGD

**Figure 4:**
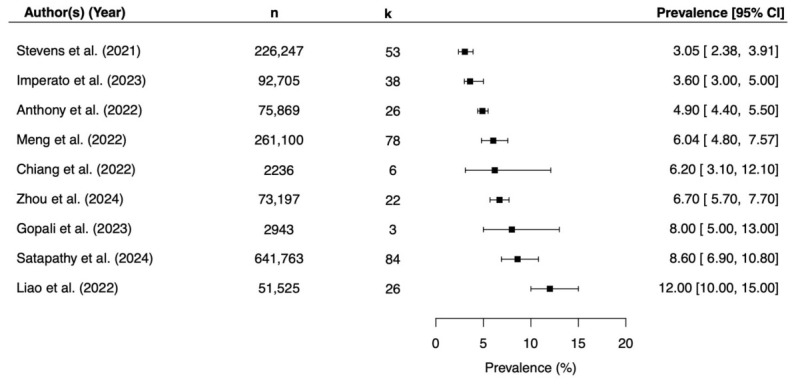
Forest plot for prevalence of IGD for each included meta-analysis [22,23,38,39,40,41,42,43,44]. *Note. n* = sample size, *k* = number of studies.

The overall median prevalence of IGD across the nine included meta-analyses was 6.20%, with an interquartile range between 4.25% and 8.30% (as shown in Figure 4). This estimate represents a simple median of the nine meta-analytic point estimates and not a statistically weighted pooled prevalence estimate. It represents a reference point for the central tendency of existing meta-analytic estimates.

### 3.4. Subgroup Analyses

We were unable to conduct subgroup analyses for time period and sampling methods because fewer than two meta-analyses consistently examined the same time frames or sampling approaches.

#### 3.4.1. Demographic Factors

**Figure 5:**
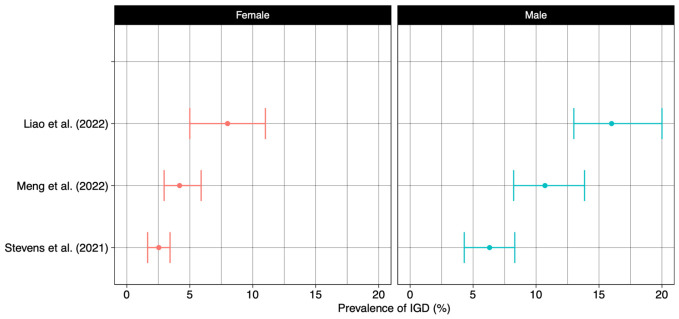
Subgroup prevalence estimates by gender [22,39,40].

Sex. Three meta-analyses examined the prevalence of IGD in males and females [22,39,40]. Figure 5 summarizes the subgroup analyses by sex. Overall, the results showed a significantly higher prevalence in males (*Mdn* = 10.17%, Range = 6.31–16.00%) than in females (*Mdn* = 4.19%, Range = 2.54–8.00%) in all three meta-analyses.

Age. Table 7 presents the three meta-analyses that examined the prevalence of IGD in adolescents and adults [23,40,44]. However, direct comparisons between adolescents and adults were limited because the meta-analyses used different age definitions for these groups, creating substantial heterogeneity. Therefore, these findings should be interpreted as inconclusive rather than as evidence that developmental differences in IGD prevalence are absent.

Future prevalence studies and meta-analyses should report exact age ranges, mean age, standard deviations, and separate estimates for children, adolescents, young adults, and adults whenever possible.

Region. Table 8 shows the four meta-analyses that reported the prevalence of IGD in the European region [22,23,40,42]; three of these meta-analyses also reported the prevalence estimates for Asia [22,23,42]. Broader regions were compared rather than individual countries because the countries analyzed varied across studies, making direct country-level comparison difficult. In addition, any apparent country differences could be confounded with study-specific methods such as sample characteristics. Regional groupings were therefore used so that findings were synthesized more consistently across reviews.

Overall, the prevalence of IGD was found to be higher in Asia (*Mdn* = 5.08%, Range = 4.00–7.50%) than in Europe (*Mdn* = 2.66%, Range = 2.10–4.27%), although this difference was non-significant in all except for one meta-analysis. One meta-analysis reported a significantly higher prevalence rate in Asia (7.50%, 95% CI = 6.30–8.60%) than in Europe (2.60%, 95% CI = 1.60–3.70%) [23].

Type of Gaming Profile. Overall, Table 9 summarizes the prevalence of IGD among gamers and the general population [39,42,44]. All three meta-analyses show a higher prevalence rate in gamers (*Mdn* = 8.20%, Range = 6.40–13.00%) than in the general population (*Mdn* = 3.10%, Range = 2.30–10.00%). However, the findings were inconsistent, with one meta-analysis reporting non-significant results [42] and two meta-analyses reporting significant results [39,44]. Specifically, in both meta-analyses, they found a significantly higher prevalence rate among gamers (6.40%, 95% CI = 5.20–7.60%; 13.00%, 95% CI = 12.00–15.00%) than in the general population (3.10%, 95% CI = 2.40–3.80%; 10.00%, 95% CI = 8.00–11.00%), respectively [39,44].

#### 3.4.2. Method Factors

**Figure 6:**
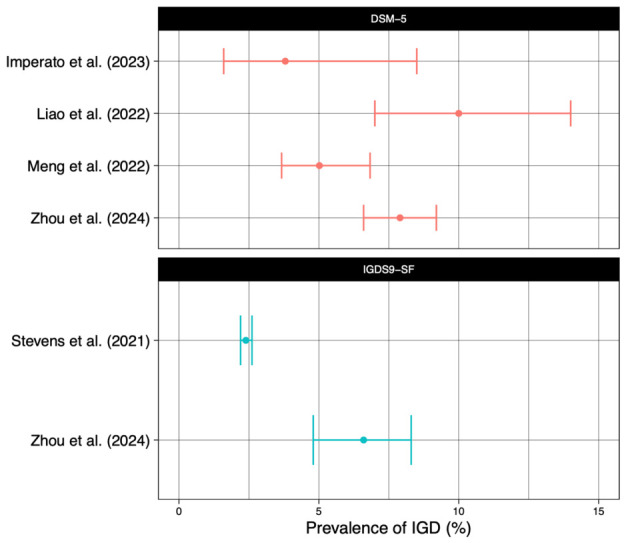
Subgroup prevalence estimates by assessment tools [22,23,39,40,42].

Assessment tools. Five meta-analyses examined the prevalence of IGD for different assessment tools, as shown in Figure 6 [22,23,39,40,42]. There were two categories of assessment tools identified. The two families consist of DSM-5 (*Mdn* = 6.48%, Range = 3.80–10.00%) and IGDS9-SF (*Mdn* = 4.50%, Range = 2.39–6.60%). Overall, we found that the prevalence of IGD tends to be slightly higher when measured by DSM-5 tools as compared to IGDS9-SF; however, in one meta-analysis, the differences between DSM-5 tools (7.90%, 95% CI = 6.60–9.20%) and the IGDS9-SF (6.60%, 95% CI = 4.80–8.30%) were found to be insignificant [23]. However, these results should be interpreted as an exploratory and partial comparison rather than a comprehensive evaluation of all measurement differences. Although additional types of scales were identified during data extraction, fewer than two meta-analyses had examined each of these measures. Therefore, they were not included in the subgroup analyses.

## 4. Discussion

This umbrella review synthesized evidence from nine meta-analyses on the prevalence of Internet Gaming Disorder (IGD). Across the included meta-analyses, the median prevalence estimate was 6.20%, with an interquartile range of 4.25% to 8.30%. Although this median is used only as a descriptive indicator of the approximate magnitude of existing meta-analytic estimates and should not be interpreted as a definitive culturally invariant prevalence rate, it suggests that IGD affects a sizeable number of individuals across the populations examined. Descriptively, a prevalence of 6.20% is roughly equivalent to one in sixteen individuals. Given the large scale of global gaming participation, even prevalence estimates within this range may represent an important public health concern.

The subgroup findings indicated that males generally showed higher IGD prevalence estimates than females. This pattern was observed across the included meta-analyses that examined gender differences. One possible explanation is that males tend to engage in gaming more frequently and for longer durations than females. For example, a study found that 50.7% of males played video games every day compared with 9.8% of females [47], while another study reported higher average weekly gaming time among males than females [48]. These differences in gaming exposure may partly contribute to a higher IGD prevalence among males. Furthermore, differences in gaming motivation may also help explain this pattern. One study suggested that males may be more strongly motivated by reward-related aspects of gaming [49], while another argued that greater reward sensitivity may contribute to the maintenance of gaming behaviors [50]. Similarly, a study suggested that boys’ gaming behavior may be reinforced by feelings of success and achievement [51]. In addition, gender differences in preferred game genres may be relevant. It was found that males were more likely to prefer action, role-playing, and sports games, whereas females were more likely to prefer puzzle games [48]. Because certain game genres may be associated with stronger reward structures, social competition, or prolonged engagement, genre preference may partly explain why males show higher IGD prevalence estimates. However, these explanations should be interpreted cautiously, as gender differences in IGD are likely influenced by multiple social, psychological, and contextual factors.

For age, the available evidence did not show consistent differences between adolescents and adults. This finding should be interpreted with caution because the included meta-analyses used different definitions of age groups, making direct comparisons difficult. Nevertheless, the lack of consistent age differences suggests that IGD may not be explained by age alone. Instead, individual differences in reward sensitivity, impulsivity, self-control, and maladaptive gaming-related cognitions may play a more important role. For instance, it was found that individuals with IGD showed stronger hedonic responses to gaming stimuli and reduced sensitivity to monetary rewards compared with controls [52]. Similarly, another study found that impulsivity, low self-control, and maladaptive cognitions were associated with IGD among adolescents [53]. These findings suggest that vulnerability to IGD may depend less on age category itself and more on psychological mechanisms that can operate across age groups. From a developmental psychopathology perspective, these mechanisms may be especially relevant during adolescence, when reward processing, peer influence, and self-regulation are still developing [54]. However, the present review cannot determine whether IGD has a different clinical course across developmental stages because the included meta-analyses did not report age-stratified findings consistently.

Regional findings were mixed. The median prevalence estimate was higher in Asia than in Europe, but only one included meta-analysis reported a statistically significant regional difference. Therefore, the present findings should not be interpreted as conclusive evidence that IGD is universally more prevalent in Asia than in Europe. Broad regional categories such as “Asia” and “Europe” also include diverse countries, cultures, gaming environments, and research practices. As such, regional comparisons may be affected by differences in sampling, assessment tools, gaming norms, and study design. Nevertheless, cultural and social factors may contribute to regional variation in IGD prevalence. In some Asian contexts, gaming and esports may be highly visible, socially embedded, and culturally normalized. For example, a study highlighted the growing legitimacy and cultural significance of esports [55]. Social motivations for gaming may also be relevant. It was also reported that Singaporean participants were more likely than Australian participants to view themselves as part of a group, and this stronger group orientation was associated with social motivations for gaming and higher IGD symptoms [56]. These findings suggest that social and cultural pathways may shape gaming engagement and IGD risk. However, future research should avoid treating regions as homogeneous categories and should examine more specific country-level, cultural, and methodological factors.

In addition, the type of gaming profile also appeared to influence prevalence estimates. Meta-analyses that compared gamers with general-population samples generally found higher prevalence estimates among gamers. However, this pattern is largely expected because gamer-only samples include individuals who actively engage in gaming, whereas general-population samples include both gamers and non-gamers. As such, these differences are likely driven, at least in part, by differences in sample composition rather than indicating inherently higher risk among all gamers. Consequently, comparisons between gamer-only and general-population samples should be interpreted cautiously, as they are not strict epidemiological comparisons. Future prevalence research should clearly distinguish between these sampling frames, as combining them may obscure meaningful differences and lead to misleading estimates.

In terms of assessment tools, because fewer than two meta-analyses reported comparable estimates for the same measure, a detailed subgroup analysis of a wider range of instruments was not possible. However, DSM-5-informed Internet Gaming Disorder measures, ICD-11-informed Gaming Disorder measures, clinician-administered assessments, and self-report inventories may yield different prevalence estimates because they differ in diagnostic thresholds, symptom criteria, impairment requirements, item counts, scoring rules, and validation across populations. These methodological differences may lead to either overestimation or underestimation of prevalence, depending on the stringency of the diagnostic criteria and scoring thresholds used. Therefore, future meta-analyses should examine these measurement differences to clarify how assessment instruments influence prevalence estimates and their interpretation.

### 4.1. Clinical Implications for Child and Adolescent Mental Health

For child and adolescent mental health services, prior evidence indicates that gaming is associated with functional impairment and neurodevelopmental changes in executive control and reward systems, rather than being adequately explained by gaming time alone [24,25,57]. Taken together with our prevalence findings, this suggests a need to further investigate these mechanisms and develop targeted interventions. Clinicians may consider sleep disruption, school decline, family conflict, impaired control over gaming, continued gaming despite harm, and comorbid symptoms such as anxiety, depression, or attention-related difficulties as indicators for further assessment. Because this umbrella review synthesized prevalence meta-analyses rather than intervention studies, these implications should be interpreted as considerations for early identification and triage rather than firm treatment recommendations, causality, clinical diagnosis, or developmental trajectories over time.

### 4.2. Limitations

This umbrella review has several limitations. First, although gray literature was searched, no eligible gray literature records were ultimately included. As a result, the evidence base consisted entirely of peer-reviewed meta-analyses. This may introduce publication bias, as studies or reviews with non-significant or less prominent findings may be less likely to appear in the published literature.

Second, there were too few existing meta-analyses to support subgroup analyses by time period and sampling method. This highlights the need for additional meta-analyses to examine whether factors such as COVID-19 and increasingly sedentary lifestyles during lockdowns have contributed to higher prevalence rates and to evaluate how different sampling methods may inflate prevalence estimates and lead to misleading interpretations. Third, the number of assessment tools that could be compared was limited because few meta-analyses focused on the same instruments. Future meta-analyses should therefore also investigate differences between DSM-5- and ICD-11-based assessment tools, given emerging evidence that these diagnostic frameworks can yield different prevalence estimates. Accordingly, prevalence estimates should also be interpreted in light of this diagnostic and measurement heterogeneity.

Fourth, the small number of included meta-analyses limits confidence in subgroup interpretations, particularly for age, region, and assessment tools. In addition, some of the included meta-analyses draw on overlapping primary data, and this redundancy may reduce the statistical independence of the synthesized prevalence estimates. Moreover, because the included meta-analyses did not consistently report the study-level information required to assess the certainty of the evidence, key factors such as risk of bias and imprecision could not be synthesized within a formal rating framework. Therefore, the overall 6.20% median and the subgroup-reported prevalence estimates should not be interpreted as definitive figures, but rather as indicative starting points for the likely range of prevalence rates.

In addition, because this umbrella review summarizes prevalence using median values across meta-analyses, this approach effectively assigns equal weight to meta-analyses of varying precision and scope. However, small-*k* meta-analyses have prevalence estimates that are more vulnerable to bias and imprecision [39,40]. Descriptively, the prevalence estimate reported by the meta-analysis with the largest evidence base (84 primary studies; 641,763 participants) was similar to that reported by the meta-analysis with the smallest evidence base (3 primary studies; 2943 participants), with prevalence estimates of 8.6% and 8.0%, respectively. Although the similarity of these estimates suggests that the overall findings are unlikely to be driven solely by smaller evidence bases, the median prevalence should still be interpreted cautiously.

Another limitation is that this umbrella review synthesizes DSM-5 Internet Gaming Disorder, ICD-11 Gaming Disorder, and related self-report-based constructs under a single umbrella term. Because these frameworks differ in diagnostic criteria and thresholds, combining them affects the comparability and interpretation of prevalence estimates. Moreover, although our criteria prioritized DSM-5- and ICD-11-informed measures, some studies used closely related constructs (e.g., gaming addiction, problematic gaming) that do not fully align with formal diagnostic frameworks. These were retained because most included studies in the meta-analyses were broadly grounded in DSM-5 or ICD-11 criteria, and excluding adjacent measures would have substantially limited the evidence base. Nevertheless, differences in measurement frameworks may introduce heterogeneity into prevalence estimates.

Furthermore, this umbrella review includes a meta-analysis [38] that focuses specifically on medical students. Although this specialized population could bias the overall summary findings, we retained this meta-analysis because it met our inclusion criteria, and our umbrella review aims to incorporate evidence from diverse populations. Regarding subgroup findings, inconsistent reporting of sample sizes across the included meta-analyses restricts our ability to derive standardized estimates, and these findings should therefore be interpreted with caution. In this umbrella review, we were also unable to systematically quantify effect sizes and were instead limited to indicating which subgroup differences reached statistical significance. Lastly, given the rapidly expanding scientific literature on this topic, future work should update the mapping of the IGD literature and present global trends in a structured format.

## 5. Conclusions

Taken together, the current review of meta-analytic evidence found an overall IGD median prevalence rate of 6.20%. The findings suggest that IGD affects a sizeable number of individuals, although prevalence estimates vary depending on population characteristics, region, gaming profile, and assessment approach. The most consistent subgroup finding was a higher prevalence among males than females. In contrast, findings for age, region, gamer profile, and assessment tool were less consistent and should be interpreted with caution. These findings highlight the importance of improving consistency in IGD prevalence research. Future studies and meta-analyses should use clearly defined diagnostic criteria, report assessment tools and cut-off scores transparently, distinguish between gamer and general-population samples, and provide more detailed subgroup information. Greater methodological consistency will improve the comparability of prevalence estimates and support more targeted prevention, screening, and public health responses for individuals at risk of IGD.

## Figures and Tables

**Table 1:** Databases and keyword search string.

Database	Keywords
EBSCOhost (ERIC & PsycINFO)	(prevalen* OR incidence OR rate* OR occurrence OR epidemiolog* OR pervasiveness) AND (gamer* OR “video gam*” OR “computer gam*” OR “internet gam*”) AND (disorder OR addict* OR excessive OR pathological) AND (“meta-analy*” OR “meta analy*” OR “quantitative synthesis” OR review)
PubMed	(prevalen* OR incidence OR rate* OR occurrence OR epidemiolog* OR pervasiveness) AND (gamer* OR “video gam*” OR “computer gam*” OR “internet gam*”) AND (disorder OR addict* OR excessive OR pathological) AND (“meta-analy*” OR “meta analy*” OR “quantitative synthesis” OR review)
Web of Science	(prevalen* OR incidence OR rate* OR occurrence OR epidemiolog* OR pervasiveness) AND (gamer* OR “video gam*” OR “computer gam*” OR “internet gam*”) AND (disorder OR addict* OR excessive OR pathological) AND (“meta-analy*” OR “meta analy*” OR “quantitative synthesis” OR review)
Scopus	(prevalen* OR incidence OR rate* OR occurrence OR epidemiolog* OR pervasiveness) AND (gamer* OR “video gam*” OR “computer gam*” OR “internet gam*”) AND (disorder OR addict* OR excessive OR pathological) AND (“meta-analy*” OR “quantitative synthesis” OR review)
ProQuest Dissertations and Theses Global	prevalence AND (“internet gaming disorder” OR “game addiction”) AND review
Google Scholar	prevalence AND (“internet gaming disorder” OR “game addiction”) AND review

**Table 2:** Agreement rates for full-text screening.

Criteria	Raw Agreement Rate	
	Trial Stage (18 Full Texts)	Post-Trial Stage (153 Full Texts)
(1) Records were included if they were written or otherwise documented in English	100.00%	99.30%
(2) Records were included if they were published within the last 12 years (i.e., 1 January 2013 to 1 January 2025) [20]	100.00%	99.30%
(3) Records were included if they were meta-analyses with a systematic search component	100.00%	100.00%
(4) Records were included if they mentioned Internet Gaming Disorder (IGD)	88.90%	96.70%
(5) Meta-analyses were included if they estimated prevalence as percentages	100.00%	97.30%
(6) Meta-analyses were included if they included studies that assessed the prevalence of clinically diagnosed disorder using relevant instruments	100.00%	78.40%

**Table 3:** Agreement rates for quality assessment.

Criteria	Raw Agreement Rate	
	Trial Stage (5 Meta-Analyses)	Post-Trial Stage (4 Meta-Analyses)
(1) Was the review question stated clearly?	100.00%	100.00%
(2) Were the inclusion criteria relevant?	100.00%	100.00%
(3) Was the search strategy appropriate?	100.00%	75.00%
(4) Were the sources used adequate?	100.00%	75.00%
(5) Were the criteria for appraising studies appropriate?	100.00%	100.00%
(6) Was critical appraisal conducted by two or more reviewers independently?	80.00%	100.00%
(7) Were there methods used to minimize error in data extraction?	100.00%	75.00%
(8) Were there appropriate methods to combine studies?	100.00%	100.00%
(9) Was publication bias assessed?	100.00%	75.00%
(10) Were the recommendations for policy and/or practice relevant?	100.00%	100.00%
(11) Were the recommendations for future research relevant?	100.00%	100.00%

**Table 4:** Agreement rates for data extraction.

Criteria	Raw Agreement Rate	
	Trial Stage (5 Meta-Analyses)	Post-Trial Stage (4 Meta-Analyses)
(1) Countries and regions covered	100.00%	75.00%
(2) Total number of studies	80.00%	75.00%
(3) Total sample size	80.00%	75.00%
(4) Assessment tools used	80.00%	75.00%
(5) Overall IGD prevalence	80.00%	100.00%
(6) Statistical model used	100.00%	100.00%
(7) Subgroup Analyses (if any)		
Gender	80.00%	100.00%
Type of gaming profile	100.00%	75.00%
Time period	80.00%	75.00%
Geographical location	80.00%	75.00%
Age	80.00%	75.00%
Measurement tool(s)	80.00%	75.00%
Sampling method(s)	100.00%	100.00%

**Table 5:** Methodological quality assessment of included meta-analyses using the Joanna Briggs Institute Critical Appraisal Tool.

Author(s)	Clear Research Question	Relevant Inclusion Criteria	Appropriate Search Strategy	Appropriate Sources Used	Appropriate Critical Appraisal	Critical Appraisal by Two or More Reviewers Independently	Methods Used to Minimize Data Extraction	Methods Used to Synthesize Data	Assessment of Publication Bias	Recommendation for Policy and/or Practice	Recommendation for Future Research	Total Score
Chiang et al. (2022) [38]	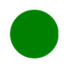	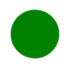	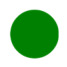	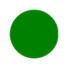	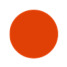	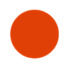	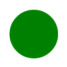	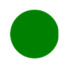	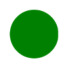	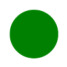	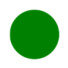	9
Liao et al. (2022) [39]	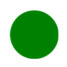	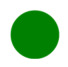	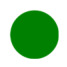	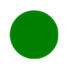	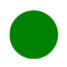	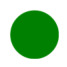	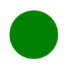	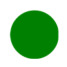	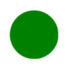	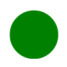	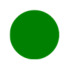	11
Stevens et al. (2021) [22]	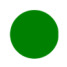	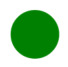	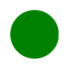	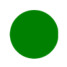	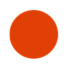	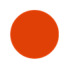	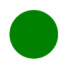	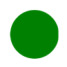	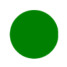	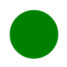	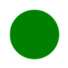	9
Meng et al. (2022) [40]	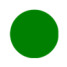	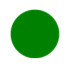	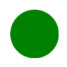	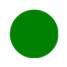	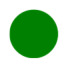	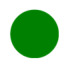	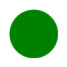	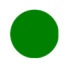	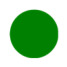	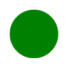	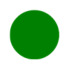	11
Zhou et al. (2024) [23]	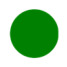	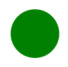	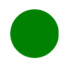	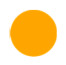	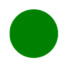	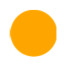	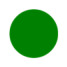	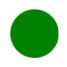	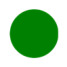	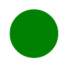	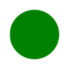	9
Gopali et al. (2023) [41]	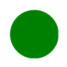	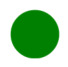	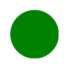	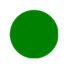	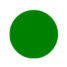	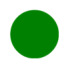	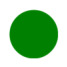	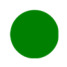	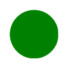	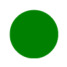	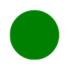	11
Imperato et al. (2023) [42]	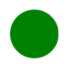	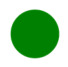	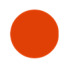	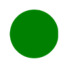	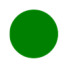	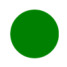	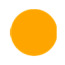	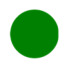	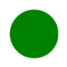	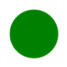	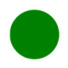	9
Satapathy et al. (2024) [43]	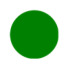	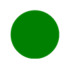	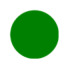	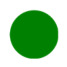	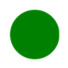	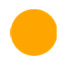	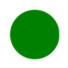	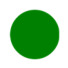	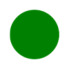	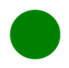	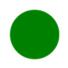	10
Anthony et al. (2022) [44]	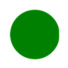	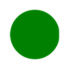	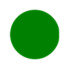	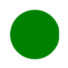	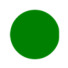	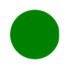	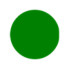	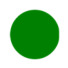	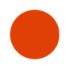	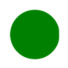	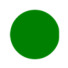	10

*Note.* Green circles represent criteria met, red circles represent criteria not met, and orange circles represent unclear or insufficient information.

**Table 6:** Characteristics of each included meta-analysis.

Author(s) (Year)	Countries and Regions Covered	Total Number of Studies	Total Sample Size	Age ^1^ (*M* or Range)	Sample Profile	Demographic	Assessment Tools Used	Overall IGD Prevalence (%)	Statistical Model Used to Synthesize the Overall Prevalence
Anthony et al. (2022) [44]	1. United Kingdom 2. Hungary 3. Netherlands 4. France 5. Germany 6. Turkey 7. Italy 8. Portugal 9. Poland 10. Finland 11. Norway 12. Croatia 13. Czech Republic 14. China 15. South Korea 16. India 17. Malaysia 18. Vietnam 19. United States 20. Brazil 21. Peru 22. Iran 23. Australia	26	75,869	Across all samples: 20.4 Adolescent samples: 14.1	Gamers, general population	Adolescents, Adults	IGDS-9a IGDS-9b POGQ IGDT-20 GAS CIUS PVP IGDT-10 IGDS9-SF IGDS-27 IGDS-9 PIGDS IGD-SF-T CSAS IGDQ PIE-9 SCI-IGD C-IGDS C-VAT IGUESS IGDS-23 IGDS IGDI C-IGDC ICMH-IGD GDS-F DISCA	4.90 (95% CI: 4.40–5.50%)	Random- effects model
Chiang et al. (2022) [38]	1. Saudi Arabia 2. India 3. Indonesia 4. Egypt	6	2236	Not specified	Medical students	Undergraduates, Postgraduates	IGD9 IGD9-SF IGAS IGDT-10 PIE-9	6.20 (95% CI: 3.10–12.10%)	Random- effects model
Gopali et al. (2023) [41]	1. Vietnam 2. Malaysia 3. Hong Kong	3	2943	Not specified	Not specified	Children, Adolescents, Adults	IGDS9-SF IGD-20	8.00 (95% CI: 5.00–13.00%)	Random- effects model
Imperato et al. (2023) [42]	1. Saudi Arabia 2. India 3. Germany 4. Turkey 5. Italy 6. China 7. Sweden 8. Vietnam 9. Israel 10. Spain 11. South Korea 12. Hungary 13. Indonesia 14. Japan 15. United Kingdom 16. Finland 17. Hong Kong 18. Nepal 19. Malaysia 20. Pakistan 21. USA 22. Brazil 23. New Zealand 24. Iraq 25. UAE	38	92,705	Across all Samples: 20.66 Undergraduate samples: 22.43	Gamers, general population	Students, Undergraduates, General population, Gamers	VASC IGDS9-SF IGD-20 VGS DSM5-IGD-9 GASA-7 SSBA GAS-7 MGUS DSM5-IGD-10	3.60 (95% CI: 3.00–5.00%)	Random- effects model
Liao et al. (2022) [39]	1. Hong Kong 2. South Korea 3. Chinese Mainland 4. Taiwan 5. Japan 6. Macao	26	51,525	Not specified	Gamers, general population	Adolescents, Young adults, Older adults	GAS-SF DSM-5 IGDS SPGA POGQ-SF CIGD IGUESS CGDS VGD-S IGDS9	12.00 (95% CI: 10.00–15.00%)	Random- effects model
Meng et al. (2022) [40]	1. African region 2. Region of the Americas 3. Eastern Mediterranean region 4. European region 5. South-East Asia region 6. Western Pacific region	78	261,100	Not specified	Not specified	Adolescents, Adults	DSM-5 DSM-IV	6.04 (95% CI: 4.80–7.57%)	Random- effects model
Satapathy et al. (2024) [43]	1. China 2. Spain 3. United States 4. Sweden 5. Netherlands 6. Finland 7. Korea 8. Germany 9. Saudi Arabia 10. Australia 11. Taiwan 12. Ireland 13. Vietnam 14. Israel 15. Tunisia 16. Lebanon 17. Egypt 18. Iran 19. Malaysia 20. Kyrgyzstan 21. Norway 22. Brazil 23. Croatia 24. Indonesia 25. Turkey 26. Slovenia 27. India 28. European Countries 29. Canada	84	641,763	Not specified	Not specified	Adolescents	AICA-S DSM-IV DSM-5 ESPAD GAS GASA GDT GSMQ-9 IAT ICD-11 IGCS IGDA IGD-10 IGD-20 IGDS IGDS9-SF IGDT-10 IGS POGQ POGQ-SF PPS SAS-SV VGDS VGEQ	8.60 (95% CI: 6.90–10.80%)	Random- effects model
Stevens et al. (2021) [22]	1. Canada 2. China 3. Germany 4. Finland 5. Hong Kong 6. Hungary 7. Iran 8. Japan 9. Lithuania 10. Multi-national 11. The Netherlands 12. Norway 13. Sweden 14. Slovenia 15. South Korea 16. Switzerland 17. Taiwan 18. United Kingdom 19. United States	53	226,247	Across all samples: 17.55	Not specified	Adolescents, Adults	A-EQ AICA-S CIUS CSAS-II GAIT GAS-7 GASA IGD IGD-9 IGDT-10 IGUESS JGSS OGCAS POGQ-SF PVP VAT YIAS-20 YDQ	3.05 (95% CI: 2.38–3.91%)	Random- effects model
Zhou et al. (2024) [23]	1. Saudi Arabia 2. Syria 3. Kuwait 4. China 5. Sweden 6. Italy 7. Jordan 8. Indonesia 9. Egypt 10. Iran 11. Malaysia 12. Russia 13. Belgium	22	73,197	Not specified	Not specified	Adolescents, Adults	IGD-20 IGDS9-SF GSMQ-9 DSM-5 IGD Checklist IGDT-10 GADIS-A	6.70 (95% CI: 5.70–7.70%)	Random- effects model

*Note.* IGDS-9a indicates the measure analyzed in a study [45], and IGDS-9b indicates the measure examined in a study [46]; POGQ = Problematic Online Gaming Questionnaire; IGDT-20 = Internet Gaming Disorder Test-20 Item; GAS = Gaming Addiction Scale; CIUS = Compulsive Internet Use Scale; PVP = Problem Video Game Playing Scale; IGDT-10 = Ten-item Internet Gaming Disorder Test; IGDS9-SF = Internet Gaming Disorder Scale—Short Form; IGDS-27 = Internet Gaming Disorder Scale (27-item); IGD-9 = Lemmens IGD Scale (9-item); IGDS-9 = Internet Gaming Disorder Scale (9-item); PIGDS = Internet Gaming Disorder Scale—Parental Version (9-item); IGD-SF-T = Internet Gaming Disorder—Short Form (Taiwanese version); CSAS = Video Game Dependency Scale (Computerspielabhangigkeitsskala; German version); IGDQ = Internet Gaming Disorder Questionnaire; PIE-9 = Personal Internet Gaming Disorder Evaluation-9; SCI-IGD = Structured Clinical Interview for DSM-5 IGD Criteria; C-IGDS = Chinese version of Internet Gaming Disorder Scale; C-VAT = Clinical Video Game Addiction Test; IGUESS = Internet Game Use—Elicited Symptoms Screen; IGDS-23 = 23-Item Internet Gaming Disorder Scale; IGDS = Internet Gaming Disorder Scale; IGDI = Internet Gaming Disorder Interview; C-IGDC = Chinese Internet Gaming Disorder Checklist; ICMH-IGD = International Child Mental Health—Internet Gaming Disorder Self-Report; GDS-F = Gaming Disorder Scale—Family; DISCA = DSM-5 Gaming Disorder Symptoms Checklist for Adolescents; IGAS = Internet Gaming Addiction Scale; VASC = Video Game Addiction Scale for Children; IGD-20 = Internet Gaming Disorder Test; VGS = Video Gaming Scale; DSM-IGD, Internet Gaming Disorder scale based on DSM-5; DSM-IGD-9 = 9-item Internet Gaming Disorder scale based on DSM; DSM-IGD-10 = 10-item Internet Gaming Disorder scale based on DSM; GASA-7 = 7-item Game Addiction Scale for Adolescents; MGUS = Maladaptive Game Use Scale; GAS-SF = Game Addiction Scale—Short Form; DSM-5 = Diagnostic and Statistical Manual of Mental Disorders, Fifth Edition; IGDS = Internet Gaming Disorder Scale; SPGA = Scale of Problematic Game Playing; POGQ-SF = 12-item Problematic Online Gaming Questionnaire—Short Form; CIGD = 11-item Chinese version of the Internet Gaming Disorder Questionnaire; CGDS = Chinese Gaming Disorder Scale; VGD-S = Video Gaming Dependency Scale; IGDS9 = 9-item Internet Gaming Disorder Scale—Short Form; DSM-IV = Diagnostic and Statistical Manual of Mental Disorders, Fourth Edition; AICA-S = Assessment of Internet and Computer Game Addiction Scale; ESPAD = European School Survey Project on Alcohol and Other Drugs; GASA = Gaming Addiction Scale for Adolescents; GDT = Gaming Disorder Test; GSMQ9 = Game Social Media Questionnaire-9; IAT = Internet Addiction Test; ICD-11 = International Classification of Diseases, Eleventh Revision; IGCS = Internet Gaming Cognition Scale; IGDA = Internet Gaming Disorder Assessment; IGD-10 = Internet Gaming Disorder Scale-10; IGD-20 = Internet Gaming Disorder Scale-20; IGS = Internet Gaming Scale; POGQ = Problematic Online Gaming Questionnaire; PPS = Perceived Problem Scale; SAS-SV = Self-Rating Anxiety Scale—Short Version; VGDS = Video Game Dependency Scale; VGEQ = Video Game Engagement Questionnaire; A-EQ = Addiction Engagement Questionnaire; CSAS-II = Videogame Dependency Scale; GAIT = Gaming Addiction Identification Test; GAS-7 = 7-item Gaming Addiction Scale; IGD = Internet Gaming Disorder; JGSS = Japanese Gaming Social Survey; OGCAS = Online Gaming Attitudes and Cognitions Scale; POGQ-SF = Problematic Online Gaming Questionnaire—Short Form; VAT = Videogame Addiction Test; YIAS-20 = Young Internet Addiction Test (20-item); YDQ = Young Diagnostic Questionnaire; GSMQ-9 = Gaming and Social Media Questionnaire; DSM-5 IGD Checklist = Diagnostic and Statistical Manual of Mental Disorders, Fifth Edition, Internet Gaming Disorder Checklist; GADIS-A = Gaming Disorder Scale for Adolescents; *M* = Mean. ^1^: Some of the individual papers analyzed in the nine meta-analyses did not include the mean age or mean age range; hence, we were unable to accurately calculate the values to give a proper representation of our findings and reported ‘not specified’ instead.

**Table 7:** Subgroup analyses for age.

Author(s) (Year)	Subgroups	*k*	Prevalence, 95% CI
Meng et al. (2022) [40]	Adolescents	40	6.94% [5.20%, 9.21%]
	Adults	33	5.05% [3.54%, 7.16%]
Zhou et al. (2024) [23]	Adolescents	10	7.10% [5.60%, 8.50%]
	Adults	12	6.50% [4.50%, 8.50%]
Anthony et al. (2022) [44]	Adolescents	–	4.90% [4.20%, 5.50%]
	Adults	–	4.80% [3.60%, 5.90%]

*Note. k* = number of studies.

**Table 8:** Subgroup analyses for geographic location.

Author(s) (Year)	Subgroups	*k*	Prevalence, 95% CI
Stevens et al. (2021) [22]	Asia	10	5.08% [3.29%, 7.78%]
	Europe	37	2.72% [1.96%, 3.75%]
Meng et al. (2022) [40]	Europe	34	4.27% [2.90%, 6.25%]
Zhou et al. (2024) [23]	Asia	19	7.50% [6.30%, 8.60%]
	Europe	3	2.60% [1.60%, 3.70%]
Imperato et al. (2023) [42]	Asia	23	4.00% [2.40%, 6.50%]
	Europe	13	2.10% [1.00%, 4.60%]

*Note. k* = number of studies.

**Table 9:** Subgroup analyses for type of gaming profile.

Author(s) (Year)	Subgroups	*k*	Prevalence, 95% CI
Liao et al. (2022) [39]	Gamers	10	13.00% [12.00%, 15.00%]
	General population	16	10.00% [8.00%, 11.00%]
Imperato et al. (2023) [42]	Gamers	3	8.20% [2.60%, 22.70%]
	General population	14	2.30% [1.40%, 4.00%]
Anthony et al. (2022) [44]	Gamers	16	6.40% [5.20%, 7.60%]
	General population	14	3.10% [2.40%, 3.80%]

*Note. k* = number of studies.

## Data Availability

Data are made publicly available at OSF (https://osf.io/6pkna/overview?view_only=9a13d4018f73453fbec2f4260b950e20).

## Supplementary Materials

The following supporting information can be downloaded at https://www.mdpi.com/article/10.3390/brainsci16070728/s1, Table S1: PRISMA 2020 Checklist.

## Abbreviations

The following abbreviations are used in this manuscript:

IGD	Internet Gaming Disorder
